# Saffron’omics’: The challenges of integrating omic technologies

**Published:** 2016

**Authors:** Sameera Sastry Panchangam, Maryam Vahedi, Mohankumar Janardhan Megha, Anuj Kumar, Kaamini Raithatha, Prashanth Suravajhala, Pratap Reddy

**Affiliations:** 1 *Bioclues.org, Kukatpally, Hyderabad 500072, Telangana, India*; 2 *Department of Horticultural Science, Faculty of Agricultural Sciences and Engineering, College of Agriculture and Natural Resources, University of Tehran, Karaj 4111, Iran*; 3 *Advanced Center for Computational & Applied Biotechnology, Uttarakhand Council for Biotechnology, Dehradun 248007, India*; 4 *Department of Applied Mathematics, the Maharaja Sayajirao University of Baroda 390002, Gujarat*

**Keywords:** *Genomics*, *Systems Biology*, *Medicinal value*, *Therapeutics*

## Abstract

Saffron is one of the highly exotic spices known for traditional values and antiquity. It is used for home décor besides serving as a colorant flavor and is widely known for medicinal value. Over the last few years, saffron has garnered a lot of interest due to its anti-cancer, anti-mutagenic, anti-oxidant and immunomodulatory properties. Integration of systems biology approaches with wide applications of saffron remains a growing challenge as new techniques and methods advance. Keeping in view of the dearth of a review summarizing the omics and systems biology of saffron, we bring an outline on advancements in integrating omic technologies, the medicinal plant has seen in recent times.

## Introduction

Saffron (*Crocus sativus* L.) is the most expensive spice and a profitable crop of the world, which is well known for its color, taste and medicinal value. The crop is being cultivated in many countries since ancient times with Iran producing approximately 95 % of the world’s saffron (Mosavi and Bathaie). It is characterized by its long, red stigmas, which contain natural carotenoid compounds such as crocin - responsible for the color(Singla and Bhat, 2011 ▶), crocetin and picrocrocin - responsible for the bitter taste along with other compounds like kaempferols (Carmona et al., 2007 ▶) and saffranal - responsible for the flavor (Rezaee and Hosseinzadeh, 2013 ▶). 

Apart from vitamins and minerals, the flower and perianth are sources of a variety of chemicals such as anthocyanins (derivatives of delphinidin and petunidin), carotenoids (zeaxanthin, lycopene and α- and β-carotenes), volatile compounds, *viz. *flavonol glycosides (kaempferol, rhamnopyranoside, rutin, quercetin, etc.) and phenolics (vanillic, syringic, gallic, caffeic and salicylic acids) that have been relatively used for medicinal purposes. For example, crocin (C_44_H_64_O_24_), the diester which is formed from the disaccharidegentiobiose and the dicarboxylic acidcrocetin (C_20_H_24_O_4_), has found universal acceptance as a phytotherapeutic drug (Frusciante et al., 2014 ▶). Saffron, as a functional spice (Kyriakoudi et al., 2015 ▶)^,^ has been used as a flavor since ancient times. Its stigmas have considerable amount of riboflavin (vitamin B2) (Schmidt et al., 2007 ▶), which also contributes to the yellow color along with the highly water soluble compound,crocin (Tsatsaroni and Liakopoulou-Kyriakides, 1995 ▶). About 50,000 years ago, saffron-based pigment was used in home décor and cave art for wall paintings in Iraq (Bathaie et al., 2014 ▶; Zargari, 1990 ▶). Despite its high cost, saffron is also used as a fabric dye. There is an increasing demand for natural plants materials and their essential oils for cosmetic purposes, such as the aqueous, ethanolic and methanolic extracts from saffron petals (Formisanoet al., 2008 ▶). Historical studies of its uses in the ancient times show that saffron was used as a perfume and as gifts in various countries like Greece and ancient Persia (Abrishami, 1997 ▶; Leffingwell, 2002 ▶).

Of late, an increasing number of studies have explored the therapeutic effects and health benefits of saffron extracts and/or its components (apocarotenoids) against an array of diseases. Saffron finds applications as an anti-depressant (Hosseinzadeh et al., 2004 ▶), anticancer (Amin et al., 2011 ▶), hypnotic (Hosseinzadeh and Noraie, 2009 ▶), anti-inflammatory (Poma et al., 2012 ▶), hepatoprotective (Omidi et al., 2014 ▶), anti-tumor (Abdullaev, 2004 ▶; Festuccia et al., 2014 ▶); aphrodisiac (Hosseinzadeh et al., 2008 ▶) agent and as a treatment for memory diseases (Ghadrdoost et al., 2011 ▶) and skin disorders (Tabassum and Hamdani, 2014 ▶). Saffron stigmas possess antioxidant and free-radical scavenging activities as its metabolites prevent lipid peroxidation and human platelet aggregation (Jessie and Krishnakantha, 2005 ▶). A study on crocetin and saffranal demonstrated that the former is more effective in inhibiting free-radical formation in male Swiss albino mice aged 6–8 weeks (Hamid et al., 2009 ▶). This crocetin effect on level of lipid peroxidation and marker enzymes in lung cancer suggests crocetin as a potent anti-tumor agent. The anti-cancer activity of saffron and crocin usually results in cell cycle arrest. In the aforementioned studies, it has been observed that some anti-tumor drugs used in the treatment of cancer show genotoxicity. In addition, reports indicated the anti-genotoxic, anti-oxidant and chemo-preventive potential of saffron against well-known anti-tumor drugs like cisplatin (CIS), cyclophosphamide (CPH), doxorubicin (DOX) and mitomycin-C (MMC) using comet assay (Chahine et al., 2015 ▶). Furthermore, saffron as an antidepressant has been used as a promising natural alternative for the treatment of mild-to-moderate depression; however, it is essential to determine the optimal dosages and duration of this treatment. In this regard, the anti-tumor effect of saffron on skin cancer was reported (Mathews-Roth, 1982 ▶). 

Aqueous saffron was shown to suppress oxidative stress in dimethylbenz[a] anthracene (DMBA) -induced skin carcinoma in mice when treated early (Das et al., 2010 ▶). Owing to its anti-oxidant properties, it can protect the central nervous system from oxidative lesion and improve learning power. Recent reviews have discussed randomized controlled trials on the effectiveness of saffron on psychological and behavioral outcomes; current human clinical evidence recommends the use of saffron for treatment of a range of pathologies, including Alzheimer's disease, age-related macular degeneration and cardiac ischaemia (Hausenblas et al., 2015 ▶; Broadhead et al., 2015 ▶). Nonetheless, the complete list of applications of saffron is beyond the scope of this review. 

**Challenges on genomics, transcriptomics, metabolomics and proteomics**

**Genomics**

Saffron is a perennial sterile plant reproducing only vegetatively using the corms. Although a lot of work has been carried out using tissue culture and hybridization (Rubio-Moraga et al., 2014 ▶; Mir et al., 2015 ▶), propagation through corms offers no or little genetic variation in the form of somatic mutations, segregation distortions, transversions, etc., which neither of them combining in a population nor bringing heritable changes due to its sterility (Agayev et al., 2009 ▶). The triploid saffron (2n=3x=24) is known to be a probable progeny of *C*. *cartwrightianus,* which contributes to two of the three genomes, while the other parental lineages remain unclear (Fernández, 2004 ▶). Detailed intraspecific chromosome variations with respect to geographical area, classification, complex cytology and morphological characteristics (corm tunics, leaves, flowers, etc.) of the *Crocus sativus*series have been reported (Saxena, 2010 ▶). The morphogenetic architecture of saffron is still an issue of debate because various studies report contradicting molecular results. However, many studies identify variations in phenotypic and phytochemical traits due to the epigenetic changes urging the immediate need for developing molecular markers to identify these variations at molecular level, which can be further exploited for the improvement of saffron (Mir et al., 2015 ▶). Also, 27 SSRs markers were evaluated on eight Iranian-cultivated saffron ecotypes and 29 wild alleles to assess the molecular variability and discriminating capacity of these markers regarding their effectiveness in establishing genetic relationships in these Crocus ecotypes (Nemati et al., 2014 ▶). More recent reports analyzed 112 accessions using Factorial Correspondence Analysis (individual level) of Amplified Fragment Length Polymorphism (AFLP) and methyl-sensitive AFLP to search for variations at the genetic and epigenetic (cytosine methylation) levels (Busconi et al., 2015 ▶). These studies indicated the presence of high epigenetic variability (33.57 % polymorphic peaks and 28 types of effective epigenotypes). Efforts are underway to prevent genetic erosion and induce genetic variability in order to develop superior varieties of saffron throughout the world.

**Transcriptomics**

 ‘Saffron omics’, an initiative of the European Cooperation in Science and Technology (COST), aims to strengthen collaborative research on developing 'omic' approaches in defining the structural organization of saffron genome, DNA fingerprinting to protect the quality and improve the genetic, chemical fingerprinting, proteomics, transcriptomics and metabolomics of this crop (http://www.saffronomics.org/). Currently, there are 6,768 saffron ESTs available at (http://www.ncbi.nlm.nih.gov/nucest/?term=%22Saffron%22), since the first set of 6,603 high quality ESTs from cDNA library of a saffron stigma were produced by D'Agostino et al., (2007) ▶ (available at http://www.saffrongenes.org). Transcriptomic and genomic studies onsaffron have received much lesser attention when compared to its potential applications in therapeutics and phytochemistry. This is probably due to the low or almost null genetic variability attributed to sterile triploid and vegetative propagation (Piqueras et al., 1999 ▶). The ESTs identified till date correspond to floral development (four MIKC type-II MADS-box cDNAs) (Tsaftaris et al., 2011 ▶), markers to detect adulteration in traded saffron (Bar-MCA analysis (Jiang et al., 2014 ▶), AS-PCR and SCAR (Shen et al., 2007 ▶; Torelli et al., 2014 ▶), environmental and pathogenic stresses (Husaini, 2014 ▶) and developmental pathways (Álvarez-Ortí et al., 2004 ▶). Similar transcriptomic studies on saffron have led to the dissection biosynthetic pathways of carotenoids (Castillo et al., 2005 ▶) and flavonoids for characterization of glucosyltransferase (Moraga et al., 2009 ▶). Additionally, deep transcriptomics analysis has identified carotenoid cleavage dioxygenase (CCD2), a novel dioxygenase which catalyzes the first step of crocin biosynthesis originating from carotenoid zeaxanthin (Frusciante et al., 2014 ▶). Although bioinformatics tools have been applied for the prediction and regulation of signaling pathways, a stringent validation using *in vitro* experiments should not be given a miss. One such immune-perspective model was deliberated with TGFβ (Kahlem and Newfeld, 2009 ▶) where successful applications of both fine-scale and network-scale informatics approaches for understanding signaling pathways were reviewed. Similarly, T and B-cell epitopes of Iranian saffron profiling were predicted using bioinformatics tools (Saffari et al., 2008 ▶).

**Metabolomics**

Metabolome is a unique collection of cellular working parts that are associated with the expression of the sequenced genomes in all living organism including bacteria, plant, animal, etc. In the recent past, metabolomic analysis proved to be an incipient tool for functional gene annotation and characterization, particularly for those genes involved in regulatory pathways. Metabolomic studies aid in identifying substrates and products of enzymes without the need for going through heterologous expression systems (Beale and Sussman, 2011 ▶). Saffron metabolomics has provided an unbiased, comprehensive qualitative and quantitative overview of its metabolites such as crocetin esters, picrocrocin, safranal, etc., elucidating their association with therapeutic and aesthetic properties (Ordoudi et al., 2015 ▶). Previous studies have identified more than 160 volatile compounds (see Table 1) using chromatography combined with spectroscopy (UV, IR, NMR) and mass spectrometry (MS) techniques (Assimiadiset al., 1998; Calsteren et al., 1997 ▶). Metabolite fingerprinting obtained using ^1^H NMR spectra and chemo-metrics was reported for the authentication of both Iranian and Italian saffron (Cagliani et al., 2015 ▶; Yilmaz et al., 2010 ▶). The insights to the structural variations in crocetin esters and picrocrocin and differentiation of sugars bound to them using ^1^H NMR method were well documented (Ordoudi et al., 2015 ▶; Ordoudi and Tsimidou, 2004 ▶). While ^1^H NMR serves as a potent tool to control saffron quality deterioration, it offers specific advantages to characterize secondary metabolites. Simultaneous identification and quantification of metabolites is necessary to understand the dynamics of the metabolome in analyzing fluxes and pathways associated with saffron. However, the major challenge remains in finding variations in biochemical pathways and metabolic networks that might correlate with the physiological and developmental phenotype of a cell and tissue. 

**Proteomics**

Identification of proteins and prediction of their structure from the amino acid sequence are challenges for researchers across different biological disciplines. Complete understanding of the biological role of proteins requires knowledge of their structure and function (Pieper et al., 2006 ▶). Although proteomics studies hold promise in characterization of both known and unknown proteins, to date, only 312 protein sequence entries are reported in GenBank: (http://www.ncbi.nlm.nih.gov/protein/?term=Saffron). Protein information provides the possibility of predicting three-dimensional structure. Proteomic analysis carried out earlier identified differentially accumulated proteins in somatic embryos of saffron, which provide insights into underlying molecular mechanisms (Sharifi et al., 2012 ▶). In addition, dearth of validated structure information for a majority of plant proteins is a major hindrance to functional annotation, evolutionary analyses and building interaction networks (Pentony et al., 2012 ▶). Although there are a plethora of tools available for the prediction and visualization of secondary and tertiary structures, detailed analyses were limited to a few selected plant gene families. For instance, UniProt hosts mere 98 protein entries for *C.sativus*, of which only 5 have been reviewed; leaving a huge scope for both *in silico *and *in vitro *studies. Presently, there is a demand for atomic-level structural refinements that can generate 3D models for use in drug screening and inferring biochemical function for these saffron proteins, especially when large template structures become available Furthermore, three crystal structures are available in protein data bank (PDB) (http://www.rcsb. org/pdb/explore/explore.do?structureId=3U8E ) which can bridge that demand in finding insights into the above-mentioned mechanisms.

**Table 1:** Chemical properties of saffron metabolites (Source: Pubchem andWikipedia

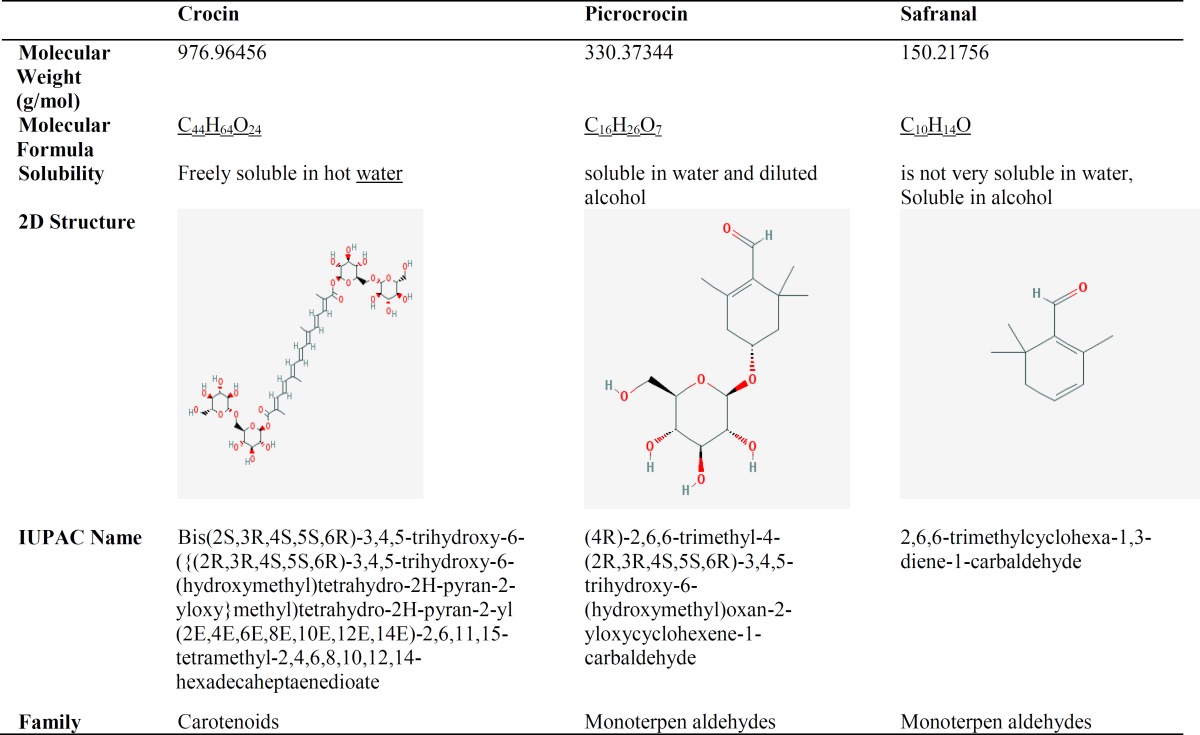

**A need for integrated Systems Biology approaches**

Over the last few years, saffron has garnered a lot of interest due to its therapeutic potential (Naghshineh et al., 2015 ▶). Integration of systems biology approaches in drug discovery has tremendous application in investigating the drug–target interaction mechanisms and in identifying novel targets in a network context (Vandamme et al., 2014 ▶; Harrold et al., 2013 ▶). 

Similarly, few studies on saffron have applied target deconvolution, reverse screening, modelling and docking for retrospective identification of molecular targets and functional components (Nithya and Shakthisekharan 2015 ▶; Bhattacharjee et al., 2012 ▶). Systems biology approaches have been applied to the non-therapeutic aspects of saffron, such as building complete metabolic pathways of the bioactive compounds, spatial-temporal expression of genes involved in clonal propagation and quantification of factors. A striking example of one such analysis was demonstrated recently (Zeraatkar et al., 2015 ▶), wherein a three-dimensional geometrical model of saffron flower was generated for the first time, using reverse engineering and laser scanning technology. The mechanical behavior of the flower could play an important role in the design of post-harvesting machinery and process. Predicting biological functions and metabolic pathways was linked to the construction of protein interaction networks (PIN) (Guan and Kiss-Toth, 2008 ▶; Wetei et al., 2013 ▶). 

*In silico* molecular dynamics and docking approach have been employed to investigate interactions between secondary metabolites of saffron (safranal, crocetin and dimethylcrocetin) and transport proteins such as β-lactoglobulin, could be valuable factors in controlling their transport to biological sites (Sahihi 2015 ▶). Reports on saffron have often highlighted the need for refining bioinformatics tools available with transcriptomic and genomic data (Fernandez and Gomez Gomez, 2005 ▶; Husaini et al., 2009 ▶; Gomez Gomez et al., 2009 ▶) but little has been done in this direction. Towards this end, the current section focuses on *in silico *approaches to build a protein interaction network of candidates involved in crocetin biosynthesis pathway. We have worked on a case study with 35 saffron protein sequences selected as a query to search for orthologs (*Oryza sativa* as reference).

 The annotation scores, based on the features are taken as per our former annotation approach (Suravajhala and Sundararajan, 2012 ▶). Sequence similarity searches were done on local FASTA (http://fasta.bioch .virginia.edu) and using BLASTp(http://blast.ncbi.nlm.nih.gov/Blast.cgi) tool against non-redundant protein sequences of *Oryza sativa*. 

**Figure 1:**
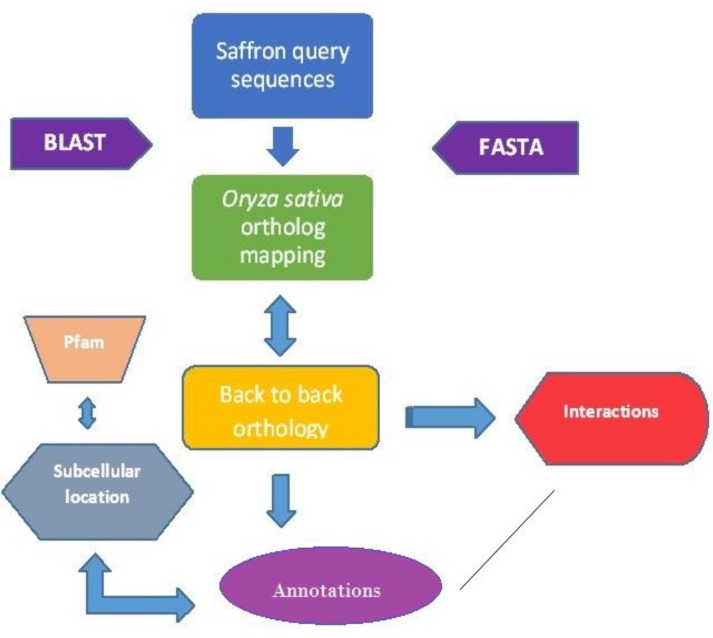
A flowchart of tools used for annotation methodology employed in obtaining the 35 protein sequences

**Figure 2:**
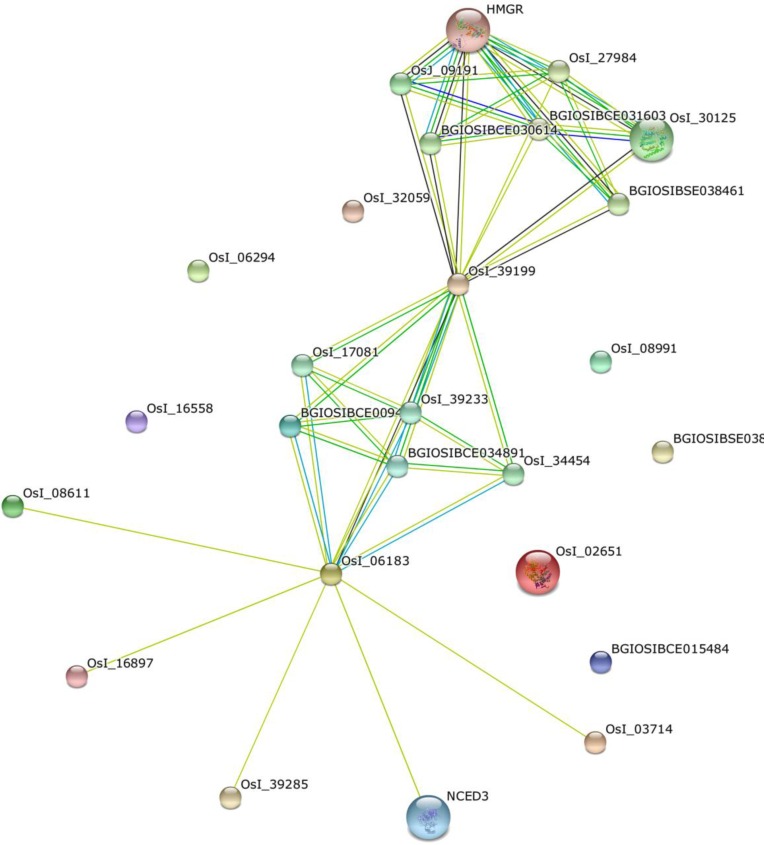
A putative protein-protein interaction map of peers of HMG Co-A reductase (HMGR) of rice interologs in saffron.Interologs are orthologous set of interacting proteins in other organisms, here saffron. The PIN was constructed using STRING database (Szklarczyk D et al. 2015) with the potential candidate proteins from the six-point scoring schema (Table 2c) as queries searched against *Oryza* as reference organism. The nodes represent the proteins which are connected by edges in the form of lines

Further characterization involving Pfam score, orthology inference, functional linkages, back-to-back orthology, subcellular location and protein associations were considered from known databases and visualizers (Figure 1). Each protein was given a value of 1 if the protein matched the classifier; else 0 was rendered (Table 2). Although only 10 out of the 35 query proteins selected have orthologs in O*ryza*, classification scoring approach revealed 15 crocetin-related proteins to have functional protein associations (Table 2). These were visualized by a protein interaction network (Figure 2) where in, interologs of three genes, *viz. *HMGR (putative 3-hydroxy-3-methylglutaryl-CoA reductase), lycopene cyclase and phytoene synthase are known to be co-expressed. These candidates that are derived from the methods employed in this analysis are concurrent with earlier reports which focused on transcriptome and metabolome experiments. It would be interesting to exploit pull-down assays and computational biology tools which could enhance our knowledge of the carotenoid biosynthetic pathway and establish other key protein interacting partners.

**Table 2:** A case study with six-point classification scoring strategy for identification of 35 protein candidate sequences. Table 2a: Identification of protein families and orthologous sequences for the query sequences from saffron

**Organism**		**Classification 1**				**Classification 2**		
**Saffron**		**Protein family scores**				**Orthology**		
**Accession**	Nomenclature	Identity	E value	Score	Score	Accession	Organism	Score
**AIF76151.1**	**UDP-glucosyltransferase UGT85U2, partial**	**UDPGT**	**2.70E-31**	**108.7**	**1**	**EAY87581.1**	***Oryza sativa***	**0**
**AIF76152.1**	**UDP-glucosyltransferase UGT85U1**	**UDPGT**	**6.00E-32**	**110.8**	**1**	**EAY87581.1**	***Oryza sativa***	**0**
**Q84KG5.1**	**Carotenoid 9,10(9',10')-cleavage dioxygenase**	**RPE65**	**5.60E-146**	**486.9**	**1**	**ABA99624.2**	***Oryza sativa***	**0**
**AIF27228.1**	**carotenoid cleavage dioxygenase 7**	**RPE65**	**2.50E-96**	**323.1**	**1**	**EAY95081.1**	***Oryza sativa***	**0**
**AIF27229.1**	**carotenoid cleavage dioxygenase 8a**	**RPE65**	**2.10E-103**	**346.5**	**1**	**NP_001044229.2**	***Oryza sativa***	**0**
**AIF27230.1**	**carotenoid cleavage dioxygenase 8b**	**RPE65**	**2.90E-103**	**346**	**1**	**NP_001044229.2**	***Oryza sativa***	**0**
**AIG94929.1**	**carotenoid cleavage dioxygenase 2**	**RPE65**	**7.40E-134**	**446.9**	**1**	**ABA99624.2**	***Oryza sativa***	**0**
**CAI60776.1**	**phytoene synthase, partial**	**SQS_PSY**	**3.60E-35**	**121.5**	**1**	**AAK07734.1**	***Oryza sativa***	**1**
**CAI60777.1**	**lycopene cyclase, partial [Crocus sativus]**	**Lycopene_cycl**	**7.00E-39**	**133.8**	**1**	**BAD16478.1**	***Oryza sativa***	**1**
**CAC95133.1**	**putative neoxanthin cleavage enzyme, partial**	**RPE65**	**5.90E-44**	**150.4**	**1**	**EAZ24320.1**	***Oryza sativa***	**0**
**ACD62475.1**	**carotenoid cleavage dioxygenase 2**	**RPE65**	**3.10E-129**	**431.6**	**1**	**ABA99624.2**	***Oryza sativa***	**0**
**ACD62476.1**	**chromoplast carotenoid cleavage dioxygenase 4a**	**RPE65**	**1.20E-106**	**357.2**	**1**	**EAZ24320.1**	***Oryza sativa***	**0**
**ACD62477.1**	**chromoplast carotenoid cleavage dioxygenase 4b**	**RPE65**	**9.10E-107**	**357.5**	**1**	**EAZ24320.1**	***Oryza sativa***	**0**
**ACM66950.1**	**flavonoid glucosyltransferase**	**UDPGT**	**1.90E-21**	**76.2**	**1**	**BAD15509.1**	***Oryza sativa***	**0**
**CAD33262.1**	**zeaxanthin cleavage oxygenase **	**RPE65**	**2.80E-83**	**280.1**	**1**	**EAZ24320.1**	***Oryza sativa***	**1**
**CAC79592.1**	**crocetindialdehyde**	**RPE65**	**5.60E-146**	**486.9**	**1**	**ABA99624.2**	***Oryza sativa***	**0**
**CAD33258.1**	**betaine aldehyde dehydrogenase, partial**	**Aldedh**	**3.40E-21**	**75.1**	**1**	**AGP76273.1**	***Oryza sativa***	**1**
**CAD70567.1**	**aldehyde dehydrogenase**	**Aldedh**	**3.90E-174**	**579.3**	**1**	**NP_001043454.1**	***Oryza sativa***	**0**
**CAC95134.1**	**putative 3-hydroxy-3-methylglutaryl-CoA reductase, partial**	**HMG-CoA_red**	**6.00E-64**	**216**	**1**	**NP_001062221.1**	***Oryza sativa***	**1**
**CAC95130.2**	**beta-carotene hydroxylase**	**FA_hydroxylase**	**3.40E-11**	**43.5**	**1**	**EEC74425.1**	***Oryza sativa***	**1**
**CAI79433.1**	**beta-carotene hydroxylase enzyme, partial**	**FA_hydroxylase**	**4.20E-07**	**30.3**	**1**	**EEC74425.1**	***Oryza sativa***	**1**
**CAI79451.1**	**beta-carotene hydroxylase enzyme, partial**	**FA_hydroxylase**	**4.20E-07**	**30.3**	**1**	**EEC74425.1**	***Oryza sativa***	**1**
**CAI79462.1**	**beta-carotene hydroxylase enzyme, partial**	**FA_hydroxylase**	**no**	**no**	**0**	**NP_001053640.1**	***Oryza sativa***	**0**
**ACD44928.1**	**plastid 9-cis-epoxycarotenoid dioxygenase**	**RPE65**	**2.30E-134**	**448.6**	**1**	**NP_001050765.1**	***Oryza sativa***	**0**
**ADA82242.1**	**lycopene beta cyclase**	**Lycopene_cycl**	**5.40E-125**	**417.1**	**1**	**BAD16478.1**	***Oryza sativa***	**0**
**Q84K96.1**	**CsZCD**	**RPE65**	**2.80E-83**	**280.1**	**1**	**EAZ24320.1**	***Oryza sativa***	**1**
**AEO50759.1**	CCD4c	RPE65	3.70E-117	391.8	1	EAZ24320.1	*Oryza sativa*	0
**AAT84408.1**	beta carotene hydroxylase	FA_hydroxylase	5.30E-07	30	1	EEC74425.1	*Oryza sativa*	1
**AAQ56280.1**	glucosyltransferase-like protein	UDPGT	1.20E-16	60.3	1	NP_001053256.1	*Oryza sativa*	0
**AAP94878.1**	glucosyltransferase 2	UDPGT	4.60E-30	104.6	1	NP_001063685.1	*Oryza sativa*	0
**Q6WFW1.1**	Crocetin glucosyltransferase 3	UDPGT	1.20E-16	60.3	1	NP_001053256.1	*Oryza sativa*	0
**Q6X1C0.1**	Crocetin glucosyltransferase 2	UDPGT	4.60E-30	104.6	1	NP_001063685.1	*Oryza sativa*	0
**CCG85331.1**	glucosyltransferase	UDPGT	7.80E-23	80.7	1	NP_001059726.1	*Oryza sativa*	0
**AAP94878.1**	glucosyltransferase 2	UDPGT	4.60E-30	104.6	1	NP_001063685.1	*Oryza sativa*	0
**CAC95132.1**	putative neoxanthin cleavage enzyme, partial	RPE65	1.10E-41	142.9	1	ABA99624.2	*Oryza sativa*	1
**CAC95131.1**	putative neoxanthin cleavage enzyme, partial	RPE65	9.80E-57	192.5	1	ABA99624.2	*Oryza sativa*	1

The first classifier (a) starts with determing if the query proteins have defined domains and is given a score of 1 if there are Pfam matches. Pfam is a protein family feature wherein we could check the presence of domains and motif family members associated with the proteins. It is followed by identifying orthologs for the queries in *Oryza* with both BLAST and stand alone FASTA. A score of 1 is given only if the queries satisfy the sequence similarity evalutioncriteon in both of the tools

**Table 2b:** Association studies for the query sequences

**Query**	**Classification 3**					**1+2+3**
	GO/association studies					
**Accession**	Gene	Annotation		Accessions	Score	
**AIF76151.1**	NA				0	1
**AIF76152.1**	NA				0	1
**Q84KG5.1**	http://amigo1.geneontology.org/cgi-bin/amigo/gp-details.cgi?gp=UniProtKB:Q8LIY8&session_id=9334amigo1418585215	carotene catabolic process		GO:0016121	1	2
**AIF27228.1**	NA				0	1
**AIF27229.1**	http://amigo1.geneontology.org/cgi-bin/amigo/gp-details.cgi?gp=UniProtKB:Q8LIY8&session_id=9334amigo1418585215	carotene catabolic process		GO:0016121	1	2
**AIF27230.1**	http://amigo1.geneontology.org/cgi-bin/amigo/gp-details.cgi?gp=UniProtKB:Q8LIY8&session_id=9334amigo1418585215	carotene catabolic process		GO:0016121	1	2
**AIG94929.1**	NA				0	1
**CAI60776.1**	NA				0	2
**CAI60777.1**	NA				0	2
**CAC95133.1**	NA				0	1
**ACD62475.1**	http://amigo1.geneontology.org/cgi-bin/amigo/gp-details.cgi?gp=UniProtKB:Q8LIY8&session_id=9334amigo1418585215	carotene catabolic process		GO:0016121	1	2
**ACD62476.1**	NA				0	1
**ACD62477.1**	NA				0	1
**ACM66950.1**	NA				0	1
**CAD33262.1**	NA				0	2
**CAC79592.1**	http://amigo1.geneontology.org/cgi-bin/amigo/gp-details.cgi?gp=UniProtKB:Q8LIY8&session_id=9334amigo1418585215	carotene catabolic process		GO:0016121	1	2
**CAD33258.1**	NA				0	2
**CAD70567.1**	NA				0	1
**CAC95134.1**	NA				0	2
**CAC95130.2**	NA				0	2
**CAI79433.1**	NA				0	2
**CAI79451.1**	NA				0	2
**CAI79462.1**	NA				0	0
**ACD44928.1**	NA				0	1
**ADA82242.1**	NA				0	1
**Q84K96.1**	NA				0	2
**AEO50759.1**	NA				0	1
**AAT84408.1**	NA				0	2
**AAQ56280.1**	NA				0	1
**AAP94878.1**	NA				0	1
**Q6WFW1.1**	NA				0	1
**Q6X1C0.1**	NA				0	1
**CCG85331.1**	NA				0	1
**AAP94878.1**	NA				0	1
**CAC95132.1**	http://amigo1.geneontology.org/cgi-bin/amigo/gp-details.cgi?gp=UniProtKB:Q8LIY8&session_id=9334amigo1418585215		carotene catabolic process	GO:0016121	1	3
**CAC95131.1**	http://amigo1.geneontology.org/cgi-bin/amigo/gp-details.cgi?gp=UniProtKB:Q8LIY8&session_id=9334amigo1418585215		carotene catabolic process	GO:0016121	1	3

If an association or linkage found through tools like Amigo2 and Genevestigator and if annotations are found for the query sequences, a score of 3 is given

**Table 2c:** Identification of interologs, sub-cellular location, interactants and potential candidates among the query sequences

**Classification 4**								**Classification 5**						**Classification 6**										**4+5+6**	**Total Reliability **
**Back to back Orthology**								**Sorting signals**						**From known databases and visualizers**											
**Accession**		Identity			E-Value	Score		TargetP	Psort				Score	Gene					Major approaches		Identity%		Score		
** AIF76151.1**		54%			0	0		other	Peroxisomes				0	OsI_08991			Os02g0755900		Neighbouhood Databases, text mining		54		0	0	1
**AIF76152.1**		54%			0	0		other	Peroxisomes				0	OsI_08991			Os02g0755900		Neighbourhood, Databases, text mining		54		0	0	1
**Q84KG5.1**		83%			0	1		other	Peroxisomes				0	OsI_39285			Putative uncharacterized protein		Neighbourhood, Databases, text mining		83		1	2	4
**AIF27228.1**		57%			0	0		mitochondria	Mitochoandria				1	OsI_16897			Os04g0550600		Neighbourhood, Databases, text mining		62		1	2	3
**AIF27229.1**		76%			0	1		other	Nucleus				0	OsI_03714			Os01g0746400		Neighbourhood, Databases, text mining		null		0	1	3
**AIF27230.1**		76%			0	1		other	Cytoplasm				0	OsI_03714			Os01g0746400		Neighbourhood, Databases, text mining		76		1	2	4
**AIG94929.1**		69%			0	1		other	Endoplasmic reticulum				0	OsI_39285			Putative uncharacterized protein		Neighbourhood, Databases, text mining		72		1	2	3
**CAI60776.1**		84%			3.00E-83	1		mitochondria	Mitochoandria				1	OsI_39199			Putative uncharacterized protein		Neighbourhood, Databases, text mining		84		1	3	5
**CAI60777.1**		79%			6.00E-72	1		other	Plasma membrane				0	OsI_06183			Os02g0190600		Neighbourhood, Databases, text mining		79		1	2	5
**CAC95133.1**		60%			7.00E-84	1		chloroplast	Chloroplast				1	OsI_08611			Os02g0704000		Neighbourhood, Databases, text mining		59		0	2	3
**ACD62475.1**		67%			0	1		other	Endoplasmic reticulum				0	OsI_39285			Putative uncharacterized protein		Neighbourhood, Databases, text mining		71		1	2	4
**ACD62477.1**		60%			0	1		chloroplast	Plasma membrane				0	OsI_08611			Os02g0704000		Neighbourhood, Databases, text mining		59		0	1	2
**ACD62477.1**		60%			0	1		other	Plasma membrane				0	OsI_08611			Os02g0704000		Neighbourhood, Databases, text mining		60		1	2	3
**ACM66950.1**		46%			7.00E-151	0		other	Endoplasmic reticulum				0	OsI_06294			Os02g0203300		Neighbourhood, Databases, text mining		46		0	0	1
**CAD33262.1**		NA				0		other	Cytoplasm			0			OsI_08611		Os02g0704000		Neighbourhood, Databases, text mining		61		1	1	3
**CAC79592.1**		NA				0		other	Chloroplast			0			OsI_39285		Putative uncharacterized protein		Neighbourhood, Databases, text mining		83		1	1	3
**CAD33258.1**		80%			5.00E-52	1		other	Peroxisomes	0				BGIOSIBCE015484			annotation not avaliable		Neighbourhood, Databases, text mining		78	1		2	4
**CAD70567.1**		79%			0	1		other	Cytoplasm	0				OsI_02651			Os01g0591300		Neighbourhood, Databases, text mining		78	1		2	3
**CAC95134.1**	86%		6.00E-91			1	other		Cytoplasm	0					HMGR	Os09g0492700		Neighbourhood, Databases, text mining		87		1		2	4
**CAC95130.2**	87%		3.00E-140			1	chloroplast		Chloroplast	1					BGIOSIBCE009468	Os03g0125100		Neighbourhood, Databases, text mining		74		1		3	4
**CAI79433.1**	90%		2.00E-52			1	mitochondria		Plasma membrane/ Mitochondria	1					BGIOSIBCE009468	Os03g0125100		Neighbourhood, Databases, text mining		90		1		3	4
**CAI79451.1**	NA		0			mitochondria	Plasma membrane/ Mitochondria		1						0	0		Neighbourhood, Databases, text mining		null		0		1	3
**ADA82242.1**	NA		0			other	Cytoplasm		0						0	0		Neighbourhood, Databases, text mining		null		0		0	0
**ACD44928.1**	72%		0			1	chloroplast		Chloroplast		1				NCED3	Os03g0645900		Neighbourhood, Databases, text mining		72		1		3	4
**ADA82242.1**	65%		0			1	other		Nucleus		0				OsI_06183	Os02g0190600		Neighbourhood, Databases, text mining		69		1		2	3
**Q84K96.1**	61%		9.00E-160			1	other		Chloroplast		0				OsI_08611	Os02g0704000		Neighbourhood, Databases, text mining		61		1		2	4
**AEO50759.1**	73%		0			1	chloroplast		Chloroplast		1				OsI_08611	Os02g0704000		Neighbourhood, Databases, text mining		74		1		3	4
**AAT84408.1**	65%		5.00E-108			1	chloroplast		Chloroplast		1				BGIOSIBCE009468	Os03g0125100		Neighbourhood, Databases, text mining		58		0		2	4
**AAQ56280.1**	NA			0		other	Peroxisomes		0						OsI_16558	Os04g0506000		Neighbourhood, Databases, text mining		38		0		0	1
**AAP94878.1**	NA			0		mitochondria	Chloroplast		0						OsI_32059	Os09g0518200		Neighbourhood, Databases, text mining		54		0		0	1
**Q6WFW1.1**	40%		4.00E-96			0	other		Peroxisomes		0				OsI_16558	Os04g0506000		Neighbourhood, Databases, text mining		38		0		0	1
**Q6X1C0.1**	48%		1.00E-144			0	mitochondria		Mitochoandria/Chloroplast		1				OsI_32059	Os09g0518200		Neighbourhood, Databases, text mining		49		0		1	2
**CCG85331.1**	52%		3.00E-173			0	other		Golgi body		0				BGIOSIBSE038738	Os07g0503300		Neighbourhood, Databases, text mining		52		0		0	1
**AAP94878.1**	NA			0		mitochondria	Chloroplast/ Mitochondria		1						OsI_32059	Os09g0518200		Neighbourhood, Databases, text mining		49		0		1	2
**CAC95131.1**	66%		7.00E-105			1	mitochondria		Mitochondria /ER		1				OsI_39285	Putative uncharacterized protein		Neighbourhood, Databases, text mining		74		1		3	6
**CAC95132.1**	82%		1.00E-99			1	Signal peptide		Chloroplast stroma		1				OsI_08611	Os02g0704000		Neighbourhood, Databases, text mining		65		1		3	6

Third classifier (2c) starts with searching for orthologs in saffron using *Oryza* as query with the help of BLAST. This is followed by assigning a sub-cellular location to the saffron query proteins, double-checked by two localization tools followed by searching for interactants using tools such as AtPID and IntAct. A consensus is reached combining all the three classifiers (Tables 2a, 2b and 2c) based on a final score >3

## Conclusions

With wide interest in increasing numbers of therapeutics, there remains a challenge in studying several non-curative compounds that could be significantly obtained from saffron. This therapeutic potential of the compounds in the form of chemotherapy or radiotherapy can allow us to find novel insights to study effects on diseases. With saffron as a chemical modulator derived from wide number of plant nutrients, employing omics technologies is the need of the hour so as to enhance the potential for drugs through possible anti-disease agents like colorants, stigmas, etc. We imagine these technologies, if integrated together can not only attribute to a better understanding of drug targets but also allow us to consider new case studies for saying ‘ome’ for medicinal plants.
